# Proof-of-concept trial in mature bulls prophylactically and therapeutically vaccinated with an experimental whole-cell killed *Tritrichomonas foetus* vaccine

**DOI:** 10.1017/S0031182025100772

**Published:** 2025-12

**Authors:** John Harvey Santos, Antonino Cavallaro, Kieren McCosker, Michael McGowan, Hannah Siddle, Loan Nguyen, Ali Raza, Gry Boe-Hansen, Ala Tabor

**Affiliations:** 1 Centre for Animal Science, Queensland Alliance for Agriculture & Food Innovation, The University of Queensland, St Lucia, QLD, Australia; 2 School of Veterinary Science, The University of Queensland, Gatton, QLD, Australia; 3 School of Environmental and Rural Science, University of New England, Armidale, NSW, Australia; 4 School of Chemistry & Molecular Biosciences, The University of Queensland, St Lucia, QLD, Australia

**Keywords:** Bovine trichomonosis, bulls, cattle, therapeutic, *Tritrichomonas foetus*, vaccine

## Abstract

*Tritrichomonas foetus* causes bovine trichomonosis, a venereal disease that reduces productivity in naturally mated cattle. Its high prevalence in Northern Australian herds underscores the need for a locally made strain-specific vaccine. This study developed and tested a whole-cell killed *T. foetus* vaccine using the Queensland isolate TfOz5 (vaccine strain) and TfOz-N36 (Northern Territory isolate) as the challenge strain. The heat-inactivated vaccine, adjuvanted with Montanide ISA 61 VG, was administered subcutaneously in 2 doses (5 × 10⁷ cells/dose) at a 1-month interval to mature bulls (*n* = 6) (4–7 years old), while controls (*n* = 6) (4–8 years old) received adjuvant with PBS. Bulls were experimentally challenged intrapreputially with live cultures of *T. foetus* at 2- and 6-months post first vaccination. A therapeutic trial with *T. foetus*-positive, persistently infected mature bulls (*n* = 10) (4–7 years old) used the same vaccine regime without the subsequent *T. foetus* challenges. The vaccine was found to be safe, causing only mild local reactions. The vaccine challenge experiment demonstrated similar duration of *T. foetus* positivity, confirmed by quantitative polymerase chain reaction (qPCR), compared to controls (94 vs. 106 days, *P =* 0.73). In the therapeutic experiment, 2/10 treated bulls tested negative for *T. foetus* at the end of the trial, while the remaining eight remained positive. Vaccinated bulls in both experiments showed significantly elevated serum anti-*T. foetus* IgG antibody levels, confirming the vaccine’s immunogenicity. These findings demonstrate that the experimental vaccine is safe and capable of eliciting a specific immune response in mature bulls.

## Graphical abstract

**Figure:**
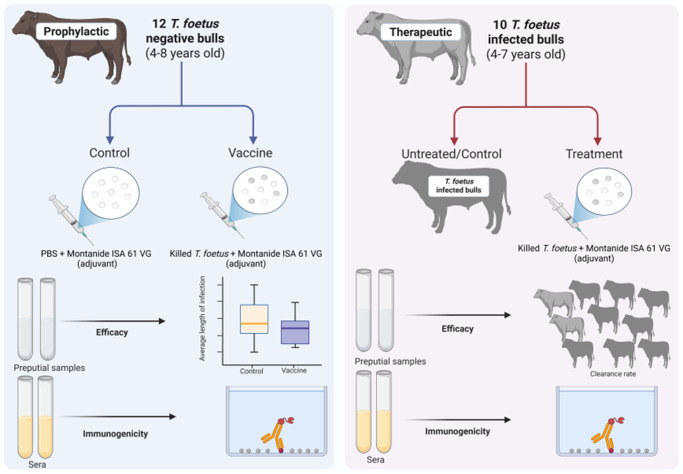

## Introduction

Bovine trichomonosis is a globally significant venereal disease of cattle, recognized as a notifiable condition by the World Organization for Animal Health (WOAH, formerly OIE). The disease is caused by the flagellated protozoan parasite *Tritrichomonas foetus*, which colonizes the epithelial surfaces of the urogenital tract – particularly the lumen and crypts of the prepuce and penis in bulls, which serve as asymptomatic carriers (Parsonson et al, 1974; Cobo et al, 2011). Transmission primarily occurs during natural mating, with infected bulls serving as the main reservoir for the parasite (Cobo et al, 2011). Unlike bulls, infected cows and heifers often exhibit clinical symptoms, including early embryonic death, vaginal discharge, pyometra, irregular oestrous cycles, and subfertility (BonDurant, 1997). The economic impact of bovine trichomonosis is substantial, resulting from reduced milk production, decreased calves weaned, and the costs associated with managing and controlling the disease (Rae, 1989; Cobo et al, 2011). Consequently, *T. foetus* poses a significant threat to cattle reproductive health and imposes considerable financial burdens on naturally mated cattle herds worldwide.

Despite being eradicated in certain regions through improved screening methods and artificial insemination, bovine trichomonosis remains a significant challenge in countries that rely on extensive grazing systems and natural mating practices (BonDurant, 1997). Evidence suggests a resurgence of bovine trichomonosis in extensive farming systems across several regions, including Australia (Calvani et al, 2021; Irons et al, 2022), South Africa (Casteriano et al, 2016), South America (Mardones et al, 2008; de Oliveira Jm et al, 2015), the United States (Ondrak, 2016; Martin et al, 2021) and Spain (Collantes-Fernández et al, 2019). To manage the disease, countries have implemented surveillance and control programmes aimed at detecting and eliminating infected bulls. This involves microscopic analysis of samples cultured from preputial scrapings or washes, followed by confirmation using polymerase chain reaction (PCR) or loop-mediated isothermal amplification (LAMP) techniques (Felleisen et al, 1998; McMillen and Lew, 2006; Oyhenart et al, 2013; Morero et al, 2021). Control strategies include testing and culling infected bulls (Yao, 2013), vaccinating cows and heifers, and enforcing a minimum of 3 months of sexual rest for infected females (BonDurant, 1997). Culling older bulls is crucial due to their higher risk of persistent *T. foetus* infection, as the parasite colonizes the epithelium of the penis and prepuce, where deeper crypts may support the growth of this microaerophilic organism (Parsonson et al, 1974; Rhyan et al, 1999).

Vaccination is a promising strategy to mitigate losses caused by bovine trichomonosis. Two commercial vaccines, Tricovac (Argentina) (Fuchs et al, 2017) and TrichGuard^®^ (USA) (Edmondson et al, 2017), have been developed using whole-cell killed *T. foetus* combined with a proprietary oil-based adjuvant and are administered subcutaneously to female cattle. Various vaccine types, including whole-cell killed, membrane antigen, and subunit vaccines, have been developed (reviewed by Santos et al. 2025). However, there is limited knowledge and literature on the efficacy of these vaccines in bulls, highlighting a significant gap in understanding their effectiveness in preventing infections and the spread of *T. foetus* (Clark et al, 1983, 1984; Herr et al, 1991; Baltzell et al, 2013; Alling et al, 2018). This gap underscores the need for further research to evaluate the potential benefits of vaccinating bulls to control bovine trichomonosis.

While these vaccines do not typically prevent infection, they effectively reduce the time needed to clear *T. foetus* from the reproductive tract, minimizing foetal loss and improving pregnancy rates by shortening the duration of infection in cows. Biosecurity and quarantine regulations restrict the importation of commercial vaccines into Australia. Therefore, developing vaccines using Australian strains of *T. foetus* is essential to meet local biosecurity requirements and effectively manage the disease. Although Australian researchers developed a vaccine for bovine trichomonosis in the 1980s, it was never commercialized due to the perceived low prevalence of the disease in southern Australian herds (Clark et al, 1983). However, recent studies have detected a high prevalence of *T. foetus* in Northern Australia in culled mature beef bulls, necessitating the urgent need for an Australian vaccine (Irons et al, 2022). The objective of this study is to develop and evaluate an Australian-sourced *T. foetus* whole-cell killed vaccine in a pilot trial focusing on mature beef bulls. This study aims to demonstrate the vaccine’s efficacy by assessing immune response and reduction in duration of *T. foetus* infection.

## Materials and methods

### *Tritrichomonas foetus* culture

#### Puification of T. foetus cultures from clinical samples

Between 2020 and 2021, a total of 66 *T. foetus* qPCR-positive isolates were collected from 20 herds across Queensland and the Northern Territory, Australia These isolates were transported in Trichomonas medium (Oxoid, ThermoFisher, Australia) at temperatures ranging from 22°C to 37°C to the QAAFI laboratories in Brisbane, Queensland, Australia. Upon arrival, isolates were incubated at 37°C for 4–5 days. Following incubation, culture pellets were resuspended in *T. foetus* freezing medium – comprising 10% foetal calf serum (ThermoFisher, Australia), 80% RPMI 1640 with L-glutamine (ThermoFisher, Australia), and 10% dimethyl sulfoxide (DMSO) (Sigma-Aldrich, Australia) – and stored at –80°C, as described by Corney (2013). Two field isolates, TfOz5 (Queensland) and TfOz-N36 (Northern Territory), were selected for use as the vaccine and challenge strains, respectively, as they were sourced from 2 different geographic locations in Australia.

To optimize growth conditions, cultures were maintained in *T. foetus* medium (TFM), prepared following the original formulations by Plastridge and Williams (1943) and Sutherland et al. (1953), and adapted according to the Australian and New Zealand Standard Diagnostic Procedures (Corney, 2013). The stored field isolates were heavily contaminated with bacteria and yeast; therefore, antimicrobial concentrations were increased and incorporated into TFM to facilitate the isolation of pure *T. foetus* seed stocks. In brief, TFM was prepared by dissolving 12.5 g/L neutralized liver extract, 5 g/L tryptose and 1.5 g/L bacto agar in deionized water, adjusting the pH to 7.4, and autoclaving at 121°C for 15 min. After cooling, 50% heat-inactivated bovine serum was added. Depending on the level of contamination, appropriate antibiotics and antimycotics were supplemented (see Table 1). Fungal and yeast contamination was assessed microscopically, while bacterial contamination was monitored using blood agar culture.

**Table 1.** Antimicrobials used to treat the heavily contaminated field *Tritrichomonas foetus* isolate cultures

Antimicrobial	Target	Supplier	Maintenance concentration, µg/mL	Range of concentrations used, µg/mL
Amphotericin B	Antifungal	ThermoFisher	1.2	0.6–4.8
Penicillin	Antibacterial	Sigma-Aldrich	1040	895–1913
Streptomycin	Antibacterial	Sigma-Aldrich	562	322–2002
Gentamicin	Antibacterial	ThermoFisher	16	8–256
Tetracycline	Antibacterial	ThermoFisher	2.5	2.5–12.5
Normacin	Broad range: Fungal Bacterial Mycoplasma	InvivoGen	200	100–400
Nystatin	Antifungal	ThermoFisher	20	20–40

#### *Mantenance of* T. foetus *cultures*

Following the establishment of pure cultures, field *T. foetus* isolates were maintained in TFM supplemented with 1040 µg/mL penicillin, 562 µg/mL streptomycin, 1.2 µg/mL amphotericin B and 16 µg/mL gentamicin. These concentrations exceed those used in standard TFM, which typically includes 750 µg/mL penicillin and 82 µg/mL streptomycin, with optional addition of 200 U of nystatin (Corney, 2013). For applications requiring liquid cultures – such as DNA extraction or preparation of vaccine and challenge strains – agar was omitted from the medium to facilitate the recovery of pure *T. foetus* cells. To maintain anaerobic conditions without compromising cell harvest, a solid agar plug was placed on top of the liquid culture medium.

## *Tritrichomonas foetus* proof-of-concept bull vaccine and treatment trial

Animal ethics approval for this study was obtained from the Production and Companion Animals UQ Animal Ethics Committee (Project Number 2021/AE000410). Two experiments were conducted: Experiment 1 comprised a vaccination trial using *T. foetus*-negative mature bulls, while Experiment 2 was a therapeutic trial involving persistently *T. foetus*-positive mature bulls. Prior to transport to the University of Queensland Pinjarra Hills Research Facility in January 2022, all animals were prescreened for *T. foetus* and *Campylobacter fetus* subsp. *venerealis* using qPCR, as described by McMillen and Lew (2006).

### Vaccine formulation

A total of 1 × 10⁹ cultured *T. foetus* cells (TfOz5) were harvested, washed with sterile phosphate-buffered saline (PBS) to remove residual culture medium and resuspended in 14 mL of 1× PBS. The suspension was heat-inactivated at 65°C for 40 min. Successful inactivation was confirmed microscopically and by the absence of growth in subsequent culture. The vaccine formulation consisted of 14 mL of the washed, inactivated TfOz5 cell suspension combined with 26 mL of Montanide ISA 61 VG adjuvant (Seppic, Australia), prepared at a 60:40 weight-to-weight ratio of adjuvant to antigen. Briefly, 26 mL of the adjuvant was homogenized (Cole-Parmer Instrument Company, USA) until a uniform cloudy emulsion was achieved. Subsequently, 14 mL of either the inactivated TfOz5 cell suspension or 1× PBS (for the control group) was slowly added to the adjuvant under continuous mixing. The final mixture was further homogenized for 3 min to ensure emulsion stability.

Vaccine (2 mL per dose, containing 5 × 10⁷ inactivated TfOz5 cells) and mock formulations (2 mL per dose, PBS + adjuvant) were dispensed into syringes and stored at 4°C until administration. Each animal received a subcutaneous (SC) injection in the neck using an 18 G needle, followed by a booster dose administered 1 month later.

### Animals

A group of 24 tropical composite bulls selected from several extensively managed commercial beef herds in Queensland, Australia were assembled for the study. The herds had a history of recent *T. foetus* diagnosis. The bulls were managed at The University of Queensland’s Pinjarra Hills Research Precinct and grazed a mixture of natural and improved tropical pastures. Initially, preputial/penile smegma samples were collected using the Tricamper device (Department of Primary Industries, Queensland Australia) from each bull on 3 consecutive occasions approximately 2 weeks apart for qPCR testing (McMillen and Lew, 2006).

### Experiment 1: Vaccine trial

Twelve tropical composite bulls, aged between 4 and 8 years (mean 5.8) which were *T. foetus* qPCR negative at all 3 tests were selected to test the efficacy of the vaccine and were divided into 2 groups: a vaccinated group (*n* = 6) which received the whole-cell killed *T. foetus* (strain TfOz5) vaccine and control group (*n* = 6) which received a mock adjuvant PBS mix. Bulls were vaccinated twice, one month apart.

**Figure 1.**
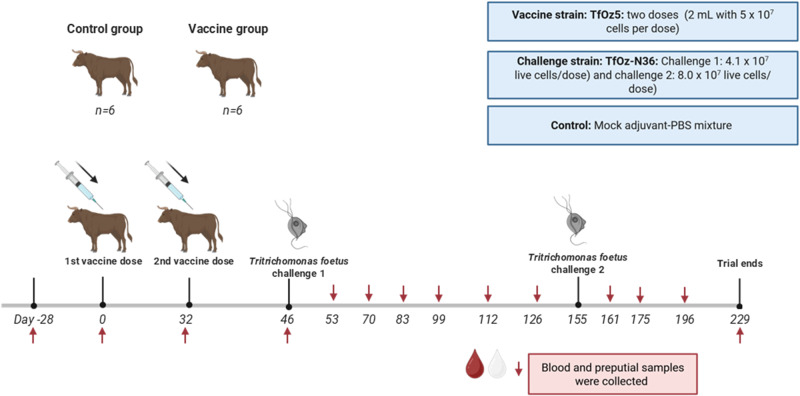
Schematic of the bull vaccine trial. Experiment 1 consists of two groups: the control (*n* = 6) and the vaccine group (*n* = 6), confirmed negative using qPCR. The vaccine group received 2 doses of the vaccine (2 ml with 5 × 10^7^ cells per dose) on Day 0 and Day 32. Both groups were challenged with the *T. foetus* TfOz-n36 strain on Day 46 (challenge 1: 4.1 × 10^7^ live cells/dose) and Day 155 (challenge 2: 8.0 × 10^7^ live cells/dose). blood and preputial samples were collected at multiple time points throughout the trial, as indicated by red arrows, and continued until Day 229.

Blood samples for *T. foetus* serology were collected via coccygeal venipuncture using 8.5 mL BD Vacutainer Serum Separator Tubes (SST II; McFarlane Medical and Scientific, Australia) using 20 G needles. Sampling was conducted at the following time points: 4 weeks prior to vaccination (Day 28), on the day of the first vaccination (Day 0), 2 weeks post-vaccination (Day 14), at the time of the booster vaccination (Day 32), and following 2 challenge exposures (Day 46 and Day 155). Additional samples were collected on Days 53, 70, 83, 99, 112, 126, 161, 175, 196 and 229 after first vaccination (Figure 1). After collection, blood samples were allowed to clot overnight, and serum was recovered by centrifugation at 2,000 × *g* for 10 min. Serum samples were stored at –80°C until further analysis. Concurrently, preputial samples were obtained using 2 Tricamper™ tools (Department of Primary Industries, Queensland, Australia) at the same time points to monitor *T. foetus* infection status by both culture and qPCR, respectively. The samples were collected while the bull was restrained in a crush and was being palpated per rectum.

#### Preparation of challenge strain and bull challenge

A *T. foetus* isolate originating from a herd different to the vaccine strain (TfOz5) was used for experimental challenge (strain TfOz-N36). The isolate was washed with sterile phosphate-buffered saline (PBS) to remove residual culture medium and resuspended in PBS. Cell viability and motility was confirmed microscopically prior to inoculation. On Day 46, 2 weeks following the sec vaccination, bulls were challenged with 3 mL of live TfOz-N36 (4.1 × 10⁷ cells/dose) delivered into the preputial cavity using a 25-inch artificial insemination catheter (Provet Pty Ltd, Australia). Following inoculation, manual preputial massage was performed for 10–15 s to facilitate distribution of the inoculum. Infection status was monitored fortnightly via *T. foetus* qPCR and culture. A second challenge was conducted on Day 155 – 5 months post-initial vaccination – using 8.0 × 10⁷ live *T. foetus* (TfOz-N36) cells per dose. Post-sec challenge, bulls were monitored monthly by qPCR and culture until the end of the study on Day 229.

Following each vaccination and challenge, bulls were clinically inspected for signs of local and systemic reactions. Injection sites were examined for swelling or abscess formation.

#### Experiment 2: Therapeutic trial

**Figure 2.**
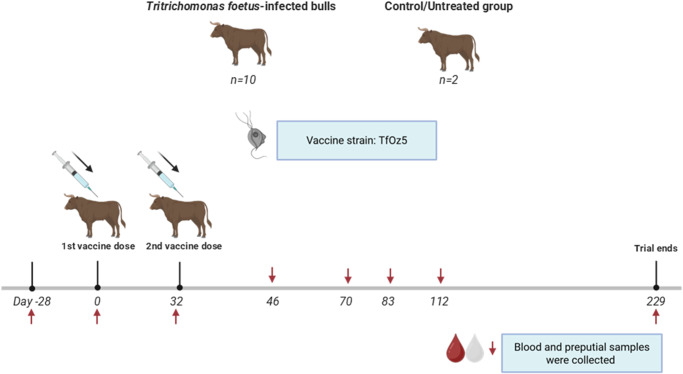
Schematic of the bull therapeutic trial. Experiment 2 consisted of 12 bulls persistently infected with *Tritrichomonas foetus*. The treatment group (*n* = 10) received 2 doses of the vaccine (2 ml containing 5 × 10⁷ cells per dose) administered on Day 0 and Day 32. The untreated control group (*n* = 2) did not receive the vaccine and was included to monitor the natural course of *T. foetus* infection and serve as a baseline for comparison. Blood and preputial samples were collected at multiple time points throughout the trial as indicated by red arrows until Day 229.

Twelve tropical composite breed bulls, aged between 4 and 7 years (mean 5.75), were confirmed *T. foetus*-positive by qPCR prior to enrolment in the therapeutic trial. Pre-screening was conducted by collecting 3 consecutive preputial samples using a Tricamper™ (as described in Experiment 1). *Tritrichomonas foetus* qPCR-positive bulls were divided into 2 groups: a treatment group (*n* = 10) which received the whole-cell killed *T. foetus* (strain TfOz5) vaccine and control/untreated group (*n* = 2) (Figure 2). The limited number of bulls in the control group was due to constraints in animal availability – only 2 suitable bulls remained for inclusion in the therapeutic trial.

The control/untreated group did not receive the vaccine to allow monitoring of the natural progression of *T. foetus* infection and to serve as a baseline for comparison. This design aimed to evaluate the vaccine’s potential as a therapeutic intervention by assessing differences in infection dynamics between treated and untreated bulls throughout the trial. The presence of *T. foetus* was monitored by both qPCR and culture throughout the study. Blood and preputial samples were collected on Days – 28, 0, 32, 46, 70, 83, 112, and 229.

## Tritrichomonas foetus *IgG ELISA*

At the conclusion of the trial, serum samples were analysed for anti-*T. foetus* IgG using an enzyme-linked immunosorbent assay (ELISA). Whole *T. foetus* (TfOz5) cells (5 × 10⁴ in 50 µL PBS) were added to each well of a Maxisorp flat-bottom plate (Nunc Maxisorp, ThermoFisher, Australia) and incubated for 4 h at room temperature to facilitate antigen binding. Plates were subsequently washed twice with 200 µL of 95% ethanol per well and air-dried. To block nonspecific binding, wells were incubated overnight at 37°C with 200 µL of PBS-T (phosphate-buffered saline containing 0.05% Tween-20) supplemented with 5% inactivated horse serum. Serum samples were diluted 1:1,000 in blocking solution, and 100 µL of the diluted samples was added to each well, followed by a 90-min incubation at 37°C.

A horseradish peroxidase (HRP)-conjugated anti-bovine IgG antibody (Sigma-Aldrich, #A5295) was used as the secondary antibody at a 1:100,000 dilution in blocking solution. A volume of 100 µL was added per well and incubated at 37°C for 30 mins. Antibody binding was visualized using a 3,3′,5,5′-Tetramethylbenzidine (TMB) substrate solution (ThermoFisher, Cat# 34021), and the reaction was stopped with 1 N HCl. Optical density was measured at 450 nm using a Multiskan FC Microplate Photometer (ThermoFisher, Australia). Negative control wells (containing only blocking solution without serum) and positive control wells (containing serum from a bull confirmed to be infected with *T. foetus*) were included on each plate to validate assay performance and ensure consistency across experimental runs.

## Tritrichomonas foetus *DNA extraction and qPCR*

*Trichomonas foetus* DNA was extracted from bull preputial samples using the MagMAX™ CORE Nucleic Acid Purification Kit (Applied Biosystems, Australia) with minor modifications to the manufacturer’s protocol. Briefly, 5 mL of the preputial sample was vortexed and transferred into a 15 mL Falcon tube, followed by centrifugation at 4,000 × *g* for 10 min at 4°C. The supernatant was discarded, and the pellet was resuspended in 200 µL of PBS. The resuspended pellet was transferred to 2 mL Eppendorf tubes, and 10 µL of Proteinase K was added. Samples were vortexed, briefly centrifuged, and incubated at room temperature for 5 min. Subsequently, samples were incubated at 95°C for 15 min at 350 rpm in a thermomixer. A lysis and binding buffer mixture (720 µL total; 350 µL lysis buffer, 350 µL binding buffer and 20 µL magnetic binding beads) was added to each sample and incubated at room temperature for 5 min. After incubation, samples were briefly centrifuged, placed on a magnetic rack for 3 min, and the supernatant was discarded.

Samples underwent sequential washes with 500 µL of wash buffer 1 and wash buffer 2. Each wash step involved vortexing for 1 min, brief centrifugation, placement on the magnetic rack for 1 min, and removal of the supernatant. Residual liquid was carefully removed while samples remained on the magnetic rack, and the beads were air-dried. For elution, 100 µL of elution buffer was added to the dried beads. Samples were vortexed for 5 min in a thermomixer, briefly centrifuged, and placed on the magnetic rack for 3 min. The purified DNA-containing supernatant was transferred to 1.5 mL Eppendorf tubes and stored at 4°C until qPCR analysis.

qPCR for *T. foetus* detection was performed using the TaqMan™ Fast Advanced Master Mix (Applied Biosystems, Australia). Each 25 µL reaction contained 12.5 µL of 2X qPCR Master Mix, 1,125 nM each of *T. foetus* forward primer TFF2 (5′-GCGGCTGGATTAGCTTTCTTT-3′) and reverse primer TFR2 (5′-GGCGCGCAATGTGCAT-3′), and 100 nM of the *T. foetus* FAM-labelled probe TRICHP2 (5′-6-FAM-ACAAGTTCGATCTTTG-MGB-BHQ-3′) as described by McMillen and Lew (2006). An internal control assay targeting bovine mitochondrial 16S rDNA was included, comprising 1,125 nM each of MTF1 forward primer (5′-AGGGATAACAGCGCAATC-3′) and MTR1 reverse primer (5′-ATCGTTGAACAAACGAACC-3′), and 125 nM of the Cy5-labelled MITGEN probe (5′-Cy5-TTTACGACCTCGATGTTGGATC-BHQ-3′) (Cawthraw et al, 2009). The reaction mixture was completed with 6.25 µL of nuclease-free water and 5 µL of extracted DNA template.

### Statistical analysis

All statistical analyses were conducted using GraphPad Prism version 9.5.1 (GraphPad Software, San Diego, CA, USA). After satisfying the assumptions of normality and homogeneity, differences between groups were compared using Students *t*-test.

## Results

### Vaccination trial

#### Vacine safety and T. foetus clearance

No adverse reactions were observed in any bulls following vaccination or challenge. Injection sites were monitored for signs of swelling, heat, pain, or abscess formation, and no abnormalities were detected throughout the study period. In the vaccinated group, 3 of 6 bulls (Bulls 1, 4 and 19) tested positive for *T. foetus* on Day 53, 1 week after the first challenge (Table 2). One vaccinated bull (Bull 1) reverted to negative status by Day 70, while the 2 other bulls (Bulls 4 and 19) remained positive. All vaccinated bulls were positive at least once after the first challenge. By Day 126, 4 vaccinated bulls accounting for 67% of the group, were negative for *T. foetus*. After the second challenge only 4 out of the 6 bulls were positive at least once. Notably, Bulls 6 and 18 remained *T. foetus*-negative throughout the last seven sampling periods following the second challenge.

In the control group, 3 bulls (Bulls 7, 28 and 31) tested positive by Day 53 following the initial challenge on Day 46 (Table 2). All control bulls were positive at least once after the first challenge, except for one (Bull 25). After the second challenge, all control bulls were positive at least once, except for 1 (Bull 12). Four control bulls (67% of the group) remained persistently positive, while an equal number of vaccinated bulls (67%) continued to test positive at Day 229.

**Table 2.** Bull vaccine trial; quantitative polymerase chain reaction (qPCR) and culture summary following vaccination

Group	Bull number	Infection status^a^														
		Days post first vaccination														
		**−28**	**0^b^**	**32^c^**	**46^d^**	**53**	**70**	**83**	**99**	**112**	**126**	**155^e^**	**161**	**175**	**196**	**229**
Control	7	−	−	−	−	+	+	+	+	+	+	+	+	+	+	+
	12	−	−	−	−	−	+	−	+	+	+	−	−	−	−	−
	23	−	−	−	−	−	+	−	+	−	−	−	−	+	+	+
	25	−	−	−	−	−	−	−	−	−	−	−	+	+	+	+
	28	−	−	−	−	+	−	−	−	−	−	−	−	+	−	−
	31	−	−	−	−	+	+	+	+	+	+	+	+	+	+	+
Vaccine	1	−	−	−	−	+	−	−	−	−	−	+	+	+	+	+
	3	−	−	−	−	−	+	−	−	−	−	+	−	+	+	−
	4	−	−	−	−	+	+	+	+	+	+	+	+	+	+	+
	6	−	−	−	−	−	−	−	+	−	−	−	−	−	−	−
	18	−	−	−	−	−	+	−	+	−	−	−	−	−	−	−
	19	−	−	−	−	+	+	+	+	+	+	+	−	−	+	+

a qPCR; (−) negative, (+) positive.

b First vaccine dose.

c Second vaccine dose.

d First *T. foetus* challenge.

e Second *T. foetus* challenge.

**Figure 3.**
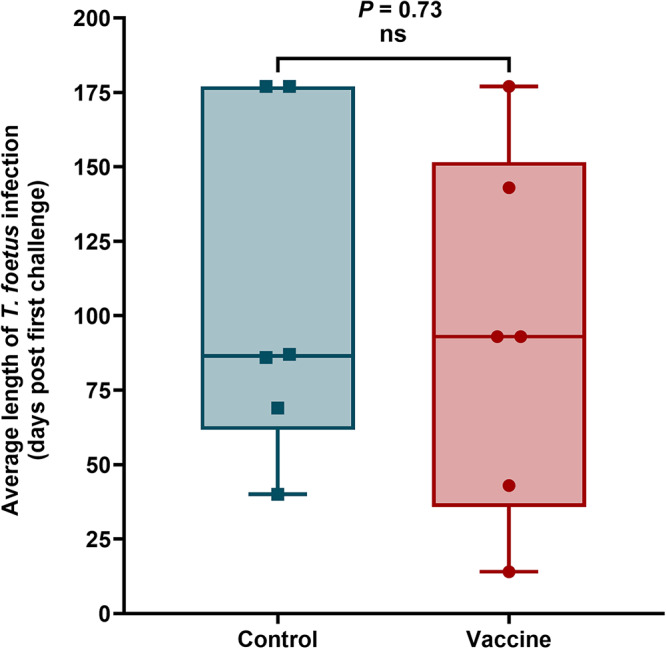
Box-and-whisker plot showing the average length of *T. foetus* infection in the bull vaccine trial. Data from each animal are presented as individual points. Horizontal lines represent median values for each group. Data were analysed using two-tailed *t*-test to compare the vaccine and control groups.

The mean duration of *T. foetus* positivity for each group was calculated as the total number of days each bull tested positive, including all periods of confirmed *T. foetus* detection, regardless of intermittent negative results. This approach provides a more accurate representation of the persistence of infection across animals. The mean duration of *T. foetus* positivity was 93.8 ± 55.2 days in the vaccinated group and 106 ± 52.6 days in the control group (Figure 3). This difference was not statistically significant (*P* = 0.73), likely due to substantial within-group variability, as reflected by the large standard deviations and individual variation among animals. Additionally, the small sample sizes in both groups may have limited the statistical power to detect modest differences in infection duration.

#### Tritrichomonas foetus-specific IgG responses

**Figure 4.**
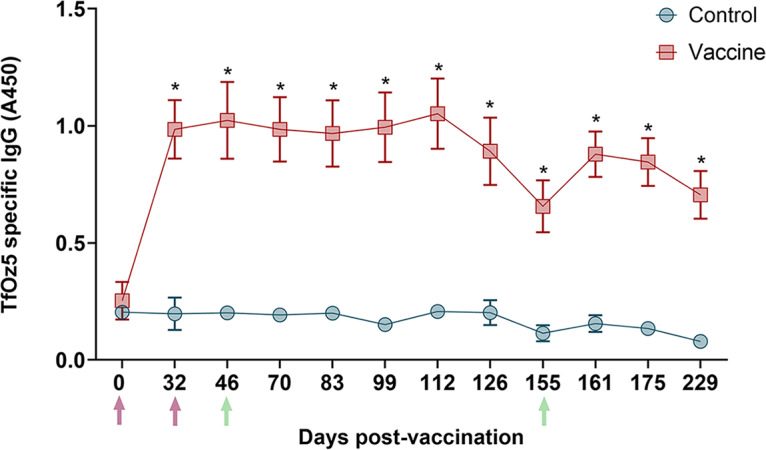
Kinetics of anti-*T. foetus* IgG levels in sera measured by ELISA. the average IgG levels measured throughout the experimental period (*x*-axis; days post-first vaccination) are represented in the graph using the ELISA absorbance reading at 450 nm (*y*-axis). error bars represent the standard deviation. purple arrows highlight the day for the first vaccination (Day 0) and second dose (Day 32). Orange arrows highlight the day of challenge 1 (Day 46) and challenge 2 (Day 155). statistically significant values in the vaccine group relative to the control group (*P* < 0.05) are denoted with an asterisk (*).

Vaccinated bulls exhibited a significant increase in IgG level at 32 days post-first vaccination (Day 32; Figure 4), compared to the control group (*P* < 0.05). IgG levels remained relatively stable through Day 112, after which a decline was observed until Day 155 following the second challenge. A subsequent increase in IgG levels was observed following this second challenge.

### Therapeutic trial

#### Tritrichomonas foetus clearance in infected bulls

Ten *T. foetus* qPCR-positive bulls in the therapeutic treatment group received 2 doses of the whole-cell killed *T. foetus* vaccine on Days 0 and 32 (Table 3). By Day 229, 2 bulls (Bulls 10 and 15) had reverted to *T. foetus*-negative status, while the remaining 8 bulls remained qPCR-positive. Although Bull 8 showed multiple negative results, it subsequently tested positive again and therefore cannot be considered to have eliminated the infection.

**Table 3.** Quantitative polymerase chain reaction (qPCR) results from the *Tritrichomonas foetus*-infected bulls therapeutically vaccinated with two doses of whole-cell killed *T. foetus* vaccine (TfOz5)

Group	Bull number	Infection status ^a^							
		Days post first vaccination							
		**−28**	**0^b^**	**32^c^**	**46**	**70**	**83**	**112**	**229**
Control ^d^	27	+	+	+	+	+	+	+	+
	29	+	+	+	+	+	+	−	+
Treatment	2	+	+	+	+	+	+	+	+
	5	+	+	+	+	+	+	+	+
	8	+	−	−	−	−	+	−	−
	9	+	+	+	+	+	+	+	+
	10	+	−	−	−	−	−	−	−
	11	+	−	+	+	+	+	+	+
	14	+	−	−	−	−	−	−	+
	15	+	−	−	−	−	−	−	−
	22	+	+	+	+	−	+	+	+
	24	+	−	+	+	−	−	−	+

a qPCR; (−) negative, (+) positive.

b First vaccination.

c Second vaccination.

d Untreated bulls.

#### Tritrichomonas foetus-specific IgG responses in infected bulls

**Figure 5.**
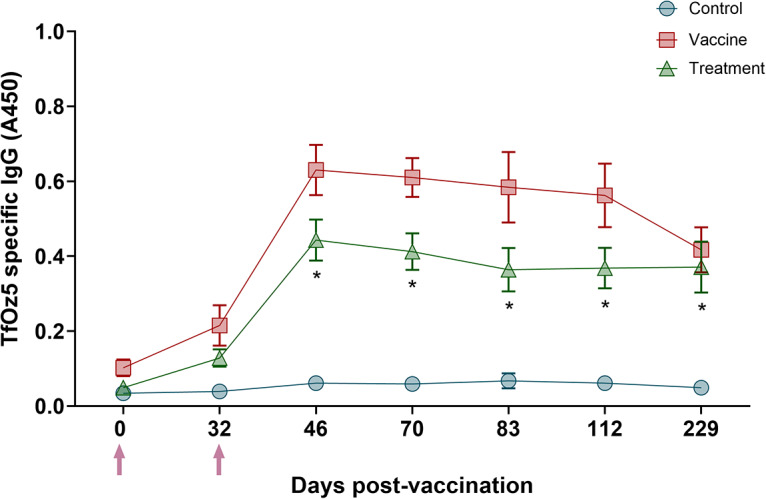
Kinetics of anti-*T. foetus* IgG levels measured by ELISA on sera in infected bulls. Vaccinations occurred at Day 0 and 32 as designated by purple arrows. Statistically significant values in the treatment group relative to the control group (*P* < 0.05) are denoted with an asterisk (*).

Bulls in the treatment group received 2 doses of the vaccine, as in the vaccine trial, but were not subjected to *T. foetus* challenge. A significant increase in IgG levels was observed in the treatment group compared to the control group at Day 46 (*P* < 0.05; Figure 5), indicating a vaccine-induced immune response in the absence of experimental challenge.

## Discussion

This study demonstrates that a whole-cell killed vaccine, developed from a locally sourced *T. foetus* isolate, elicited a robust immune response, and is safe for use in mature bulls – addressing a critical gap in trichomonosis control strategies for Australia. However, the small sample size used in this proof-of-concept study limited conclusion of the efficacy of the trial vaccine. This limitation was primarily due to logistical and welfare constraints associated with conducting intensive experimental studies of adult cattle. Despite this limitation, the findings of this proof-of-concept study are encouraging and provide valuable preliminary evidence to support further investigation – offering a targeted vaccination approach to enhance herd health, improve reproductive efficiency and support more sustainable trichomonosis management strategies within the Australian beef industry.

Currently, no vaccines are specifically approved for use in bulls. Although commercial vaccines such as TrichGuard^®^ and Tricovac are available internationally, they are primarily administered to female cattle and offer only partial protection against *T. foetus*. The absence of bull-targeted vaccines remains a significant gap, particularly given the role of mature bulls as long-term asymptomatic carriers that contribute to sustained transmission within herds. This is particularly relevant for northern Australia’s extensive cattle production systems, where *T. foetus* remains a significant challenge and prevalence rates can reach approximately 15% in some bull populations (Irons et al, 2022).

## *Eficacy and safety of whole-cell killed* T. foetus *vaccine*

Mature bulls were used to assess the safety, immunogenicity, and efficacy of the TfOz5 vaccine. No adverse reactions or clinical signs were observed following vaccination, suggesting that the vaccine can be considered safe for use in bulls. Vaccine efficacy was primarily evaluated based on the duration of *T. foetus* infection, with a similar infection rate observed between the control and vaccinated groups (67%). Although the small sample size limited the statistical power of the study, the results provide preliminary evidence supporting the potential of vaccination to reduce *T. foetus* infection in mature bulls.

A study by Clark et al. (1983) similarly investigated the use of a whole-cell killed *T. foetus* var. *Brisbane* vaccine in older bulls (3–8 years; mean 5.25). In that study, persistent infection was observed in only 25% (2/8) of vaccinated bulls, compared to 87.5% (7/8) in unvaccinated controls. The 2 vaccinated bulls that became persistently infected were 5 and 8 years old (Clark et al, 1983). While differences in vaccine formulation between the 2 studies preclude direct comparisons, both findings support the potential role of vaccination in reducing *T. foetus* infection in mature bulls.

Importantly, the vaccine elicited a robust serum IgG response, particularly after the second dose, with a further significant boost observed following *T. foetus* challenge This response underscores the vaccine’s ability to stimulate systemic immunity in mature bulls. Although the vaccine elicited a robust serum IgG response, particularly after the second dose and following *T. foetus* challenge, it remains unclear whether this response alone is sufficient for protection. It is possible that a local mucosal immune response is required for effective clearance, and that high serum IgG titres may not directly correlate with antibody functionality. For instance, agglutination assays may reveal limited activity despite elevated titres. This has been demonstrated by Hodgson et al. (1990), where monoclonal antibodies (TF3.8 and TF3.2) against *T. foetus* showed strong ELISA reactivity but failed to induce agglutination or bind surface epitopes, indicating that not all antibodies with high titres are functionally protective. Further studies are warranted to assess the functional properties of the induced antibodies and the contribution of local immunity to protection. Previous studies in cows have demonstrated that elevated genital IgA and systemic IgG levels are associated with reduced infection duration (Gault et al, 1995; Fuchs et al, 2017; Ortega-Mora et al, 2022). In vitro studies further support a functional role for IgG in *T. foetus* neutralization, including inhibition of parasite adherence and complement-mediated killing (Aydintug et al, 1993; Corbeil et al, 1989). However, the role of IgG in bulls during *T. foetus* infection remains poorly understood. Clark et al. (1983) reported that vaccination increased serum agglutinin titres, with vaccinated bulls exhibiting significantly higher titres (160–640) one month after the third dose, compared to controls (0–10). Notably, their study included bulls aged 3–8 years, and younger bulls (≤5 years) showed stronger immune responses and lower infection rates than older bulls (>5.5 years). These findings suggest that while vaccination can enhance immune reactivity in bulls, its efficacy may be influenced by age, with younger bulls likely deriving greater benefit.

## *Thrapeutic efficacy of the whole-cell killed* T. foetus *vaccine in infected bulls*

In addition to its prophylactic application, the vaccine was evaluated for potential therapeutic efficacy in bulls already infected with *T. foetus*. Previous studies on therapeutic vaccination have reported mixed results, with no definitive evidence of effectiveness in clearing established infections (Clark et al, 1984; Herr et al, 1991; Alling et al, 2018). In the present study, 20% of the vaccine treated bulls tested negative for *T. foetus* by the end of the trial. As 8 of the 10 bulls remained positive, we conclude that vaccination cannot reliably be used to treat *T. foetus* positive bulls. It is also uncertain if the 2 which did clear *T. foetus* infections following vaccination was the result of vaccination or whether they had naturally cleared the infection.

A significant challenge in controlling bovine trichomonosis is the role of older bulls as asymptomatic carriers. Consistent with previous findings (Rae et al, 2004; Michi et al, 2016), bulls over 3 years of age exhibit reduced natural resistance to *T. foetus* infection, contributing to disease persistence within herds. In a longitudinal study conducted by Rae et al. (2004), 1,984 beef bulls from 59 herds in Florida were surveyed over 24 months using preputial scrapings and culture to detect *T. foetus*. Their results demonstrated a significant association between bull age and infection status, with bulls older than 3 years more likely to be colonized by the parasite. This age-related susceptibility is likely attributable to a combination of factors, including diminished localized immune function and anatomical changes in older bulls, such as the development of deeper preputial crypts that may promote *T. foetus* colonization. Moreover, considerable variation in infection outcomes among therapeutically vaccinated bulls further complicates diagnosis and management. Consequently, older bulls not only face a heightened individual risk but may also serve as chronic reservoirs , contributing to the sustained transmission of *T. foetus* within herds.

Importantly, the robust serum IgG response observed in therapeutically vaccinated bulls – comparable to that in prophylactically vaccinated animals – indicates that the vaccine effectively stimulates adaptive immunity in mature bulls, irrespective of pre-existing infection status. However, vaccination is not successful in clearing the *T. foetus* parasites in persistently infected mature bulls. This suggests that younger bulls should be vaccinated prior to exposure to *T. foetus* and before the commencement of breeding programmes to minimize the risk of *T. foetus* herd infections.

### Recommendations and future directions

Future research should prioritize larger-scale studies to robustly validate these preliminary findings. Investigations assessing long-term immune responses, re-infection rates and vaccine efficacy in younger bulls (1–2 years old) are warranted. Such studies will provide a more comprehensive understanding of the vaccine’s performance and support its potential adoption within the Australian beef industry.

## Conclusion

The TfOz5 whole-cell killed *T. foetus* vaccine elicited robust IgG responses in mature bulls and was well tolerated, with no adverse effects observed. While the vaccine demonstrated immunogenicity, no statistically significant difference in the duration of *T. foetus* infection was observed between vaccinated and control animals. Therefore, its potential for prophylactic use requires further investigation in larger-scale studies. Although only 10 bulls were used in the therapeutic experiment, vaccination did not consistently clear *T. foetus* infections in persistently infected mature bulls. Future research should evaluate vaccine efficacy in younger bulls (1–2 years old). From a disease management perspective, this approach may help reduce the prevalence of *T. foetus* in endemic regions in Australia, particularly where testing and culling are not economically viable.
